# S77. Proffered paper: In vitro induced response patterns of antileukemic T-cells – characterization by combination of functional assays, spectratyping and next generation sequencing

**DOI:** 10.1186/2051-1426-2-S2-I15

**Published:** 2014-03-12

**Authors:** S Reuther, P Krell, FR Schuster, C Grabrucker, A Liepert, T Kroell, HJ Kolb, A Borkhardt, R Buhmann, H Schmetzer

**Affiliations:** 1 University of Munich, Med. III Hematopoetic Transplantations, Munich, Germany; 2 University of Duesseldorf, Department of Pediatric Oncology Hematology and Immunology, Duesseldorf, Germany

## Background

T-cell receptor (TCR) diversity is characterised by somatic alterations in the complementary determining region 3 (CDR3) of the human TCR β-chain. Complemented with the TCR alpha-chain, TCR diversity can hypothetically result in up to 10^18^ different TCR molecules.

Myeloid leukaemic cells can be induced to differentiate into leukaemia-derived dendritic cells (DC_leu_) regaining the stimulatory capacity of professional DCs while presenting the whole leukaemic antigen repertoire.

Our aim was to identify TCR Vβ-chain-rearrangements in T-cells stimulated with leukaemic blasts and DC_leu_ in 3 patients with AML and furthermore to detect, amplify or monitor T-cell clones with defined Vβ-profiles in correlation with antileukaemic function, in vitro and in vivo.

## Material and methods

HLA matched or HLA haplo-identical (allogeneic) donor- or autologous T-cells were repeatedly stimulated, either with leukaemic blasts or the corresponding DC_leu_ from 3 different AML-patients. Cytotoxicity assay was carried out for measuring the lytic activity of effector T-cells, spectratyping was performed to identify the restriction of the TCR Vβ-repertoire in unstimulated and stimulated T-cells and Sanger sequencing to analyse the β-chain sequence information including the CDR3 regions. Additionally next generation sequencing (NGS) was established to analyse the accurate TCR sequence information of thousands of TCR β-chains with high coverage.

## Results

No significant differences in T-cell proliferation were observed. The T-cell mediated cytolytic response patterns showed blast lysis (n=1) and blast proliferation (n=2).

Spectratyping revealed a remarkable TCR Vβ-restriction of the CD4^+^- or CD8^+^-TCR repertoire of blast- or DC/DC_leu_-stimulated T-cells, independently of blast or DC/DC_leu_ used as stimulators. Although in absolute terms, DC/DC_leu_ stimulation induced the highest grade of restriction in the CD8^+^ T-cell subset, the CD4^+^ T-cells seemed to be relatively more affected.

In vitro stimulation with DC/DC_leu_ resulted in an identical TCR (β-chain restriction pattern) as identified in vivo in a patient sample 3 months after allogeneic stem cell transplantation (SCT).

## Conclusion

A combined strategy using spectratyping and NGS with functional tests may provide useful information about the specificity and efficacy of the intra-individual variable induced T-cell response.

Spectratyping allows the identification of a restricted Vβ-repertoire by Gaussian-like distribution, NGS allows sequencing of TCR repertoires with high coverage, novel software allows the analysis of the exact length and sequence composition (the combination of the Vβ- and Jβ-genes) of the β-chains, especially of the CDR3, and the exclusion of non-functional transcripts.

The identification of defined Vβ-T-cell clones may lead to selection procedures generating Graft-versus-Leukaemia reaction- but not Graft-versus-Host disease- mediating T-cells for adoptive immunotherapy after SCT.

